# Localized Surface Plasmon-Enhanced Electroluminescence in OLEDs by Self-Assembly Ag Nanoparticle Film

**DOI:** 10.1186/s11671-015-1176-9

**Published:** 2015-12-02

**Authors:** Xiaoxiao He, Wenjun Wang, Shuhong Li, Qingru Wang, Wanquan Zheng, Qiang Shi, Yunlong Liu

**Affiliations:** Shandong Key Laboratory of Optical Communication Science and Technology, School of Physical Science & Information Technology of Liaocheng University, 252059 Liaocheng, Shandong Province People’s Republic of China; Institute des Sciences Moléculaires d’Orsay ISMO—CNRS, Université Paris-SudBât. 350, 91405 Orsay cedex, France

## Abstract

We fabricated Ag nanoparticle (NP) film in organic light emission diodes (OLEDs), and a 23 times increase in electroluminescence (EL) at 518 nm was probed by time-resolved EL measurement. The luminance and relative external quantum efficiency (REQE) were increased by 5.4 and 3.7 times, respectively. There comes a new energy transport way that localized surface plasmons (LSPs) would absorb energy that corresponds to the electron-hole pair before recombination, promoting the formation of electron-hole pair and exciting local surface plasmon resonance (LSPR). The extended lifetime of Alq3 indicates the existence of strong interaction between LSPR and exciton, which decreases the nonradiative decay rate of OLEDs.

## Background

Organic light emission diodes (OLEDs) have been widely used in TV, mobile phone, and lighting source because of their unique advantages such as flexibility, high brightness, low power consumption, and wide view [1–3]. Excellent progress has been achieved in the past few years for improving the quality of OLEDs [4–7]. For example, nearly 100 % of inner quantum efficiency can be obtained using phosphorescence [8, 9] and delayed fluorescence [10–13] materials. However, the delayed fluorescence depends heavily on the depositing conditions and the quantum efficiency is usually much lower than 100 %. Phosphorescence also has some disadvantages such as lighting impurity, unstability, and so on. In order to design OLEDs with better performance, the quantum efficiency of fluorescence needs to improve by using new technology and theory. There are many efforts attempted to break the limitation of 25 % quantum yield, which is due to spin forbidding. Surface plasmon resonance (SPR) [14–18] and localized surface plasmon resonance (LSPR) [19–22] associated with metal film and nanoparticles (NPs) have become the most attractive methods to enhance electroluminescence (EL) of OLEDs. The enhanced factor for photoluminescence (PL) can reach to as high as 10^10^ theoretically.

Many works have been done to improve the quantum efficiency of electroluminescence (EL) for inorganic materials, and the EL intensity was enhanced based on SPR and LSPR [23–25]. EL enhancement based on LSPR is difficult for OLEDs due to the damage of H^+^ and O^2+^ organics in air [26]. OLEDs need to be fabricated in a high-vacuum system to protect the device from air and water. Zhang et al. have successfully introduced the Ag NP film to OLEDs by using periodical drape metal and achieved considerable enhancement [27]. The enhancement by SPR and LSPR was attributed to the increase of the radiative decay rate while suppressing the nonradiative decay rate of excitons. However, before the forming of an exciton, electron-hole pair would relax to metastable states, which would release much energy. If the energy is effectively used, the efficiency of OLEDs may be significantly improved. In addition, because of the method they used in that work, impurity of the NPs cannot be avoided. Here, we report a study to deposit Ag NP film in OLEDs without introducing impurity and to attempt to make the best use of the energy from the electron-hole pair and exciton.

## Method

### Samples

The OLEDs were fabricated by using organic evaporating system with the pressure less than 1.0 × 10^−4^ Pa. To investigate the interaction between Ag NP film and emitting material, we choose the typical and high-efficiency organic materials. The organic layers, NPB (*N*,*N*′-diphenyl-*N*,*N*′-bis(1-naphthyl)-(1,1′-biphenyl)-4,4-diamine, hole transfer layer, 40 nm), Alq3 (tris-(8-hydroxylquinolone)aluminum, emission layer, 30 nm), and PBD (2-(4-tert-butylphenyl)-5-(4-biphenyl)-1,3,4-oxadiazole, electron injection layer, 3 nm) were deposited on ITO glass. Then, the Al film was evaporated to the top for cathode.

The organic layers were deposited at a slow rate (0.06 nm/s) to form a nanostructure. Before the continuous film forming, the cores appear first. Then, the cores that connect with each other become the integrated film. When evaporation was slow, the kinetic energy was smaller than the attraction between two organic molecules. They will be tied together. The connection of cores would be limited and form the nanostructure. Then, the Ag film was deposited on the organic layer with a much slower rate (0.01 nm/s) by laser molecule beam epitaxy system (L-MBE). Ag atom was small and slow enough to be attracted by the relatively big organic molecules. Ag NP film was deposited on electron transfer layer (PBD).

**Fig. 1:**
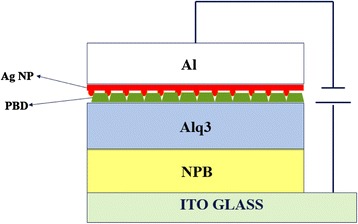
The structure of OLED with Ag NP film

Figure 1 shows the schematically structure of our OLEDs. The Ag NP film self-assembly formed in the device with Ag nanopeak embeds the organic layer.

### Measurements

#### Thickness Measurements

The accurately controlling of thickness of each layer is important to the performance of OLEDs. We use two methods to determine the thickness of films. Spectroscopic ellipsometry (M-2000VI J.A. Woollam) and AlphaStepD-100 were used to join measure thickness. Then, the thickness of the layers is controlled by using a quartz crystal monitor in the evaporating process. The morphology of films was presented by using atomic force microscope (AFM, NT-MDT Prima).

#### EL and Current Intensity Measurements

The current density-voltage and photon count intensity-voltage characteristics of the devices were measured by using a Keithley 2400 (Keithley instruments Inc.) and FLS920 (Edinburgh Instrument). The absorption spectra was measured in the wavelength range of 300–900 nm with a UV-vis spectrophotometer (U-3310 UV-vis HITACHI). The decay kinetics was measured by FLS920. All measurements were done at room temperature under ambient air.

## Result and Discussion

**Fig. 2:**
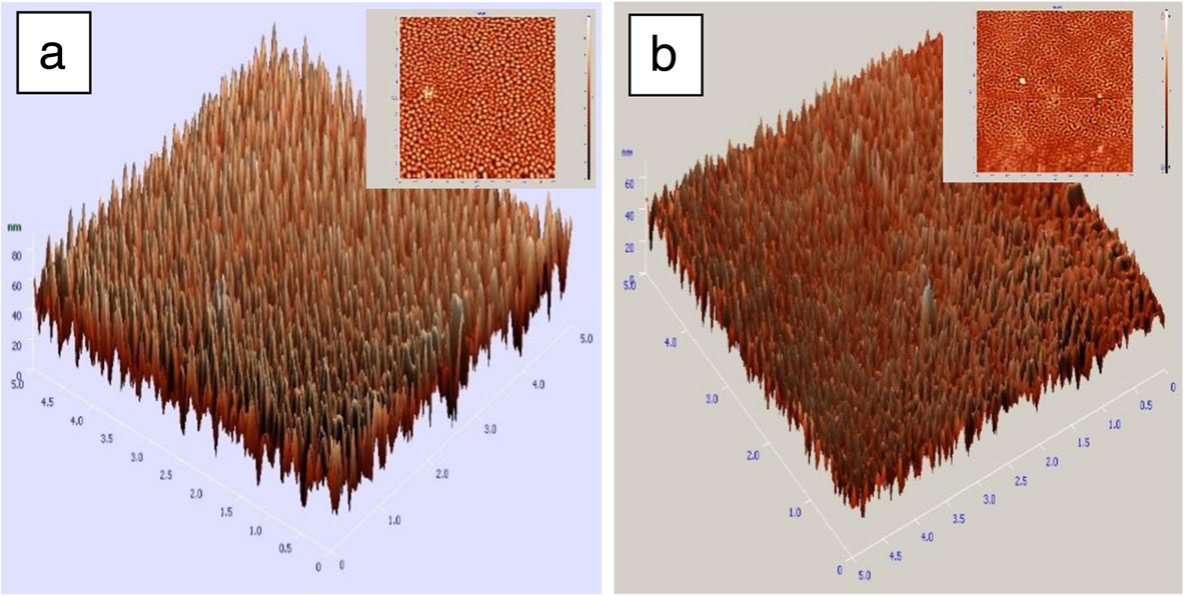
The AFM image of organic layer before (**a**) and after (**b**) deposition Ag

Figure 2 shows the AFM of films with and without Ag film. Figure 2a presents the film with uniform nanostructure peaks by controlling the evaporation rate of organics. The 3D and 2D images indicate the presence of sharp nanopeak structures, which are ~40 nm in height and ~80 nm in diameter. The depositing of Ag decreased the height of peaks from 40 to 30 nm as shown in Fig. 2. The space among the nanopeak was filled by Ag NPs.

**Fig. 3:**
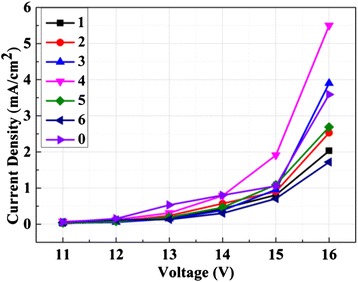
The current density of devices with different thickness of Ag NP film

Then, we fabricated the OLEDs with different thickness of Ag NPs film. Figure 3 presents the current density of the devices with different thickness of Ag NP film. It shows that current density decreases after the insertion of Ag NP film except the one with 4-nm Ag NPs. This is because thinner Ag NP film do not form the integrated film yet, which blocked electron flowing. After the Ag film increased to 4 nm, the integrated film was formed. Combining with the Al, it becomes the Ag-Al complex cathode, which increased the injection of electron. If we keep increasing the thickness of Ag film, we can observe strong LSPR in the Ag film with nanostructure.

The strong LSPR and the gathering of electron can damage the organic film, leading to the decrease of the current density.

**Fig. 4:**
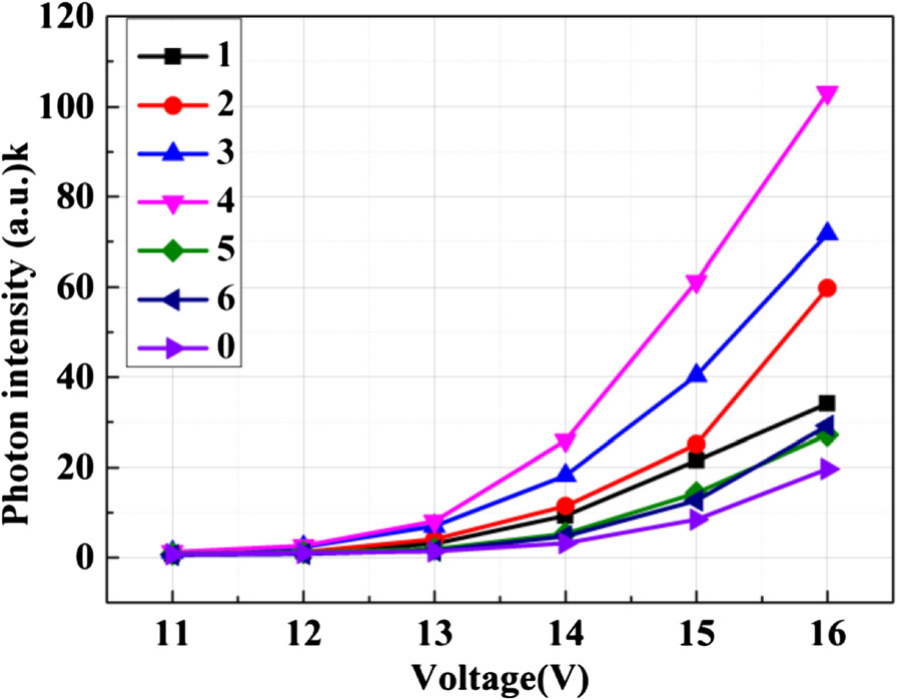
The photon intensity of devices with different thickness of Ag NP film

We then measured the photon intensity of the devices with different thickness of Ag NP film using single photon counting technique (FLS920). As showed in Fig. 4, the photon intensity of the devices increased after inserting the Ag NP film. With the thickness of Ag NP film increasing, the photon intensity of the devices increased. The device with 4-nm Ag NP film performed the largest photon intensity. When the thickness of Ag NP film is thicker than 4 nm, the devices could not supply enough carriers for combination. Furthermore, when the thickness is more than 4 nm, it may damage the devices. Thus, we chose 4 nm to be the optimal thickness of Ag NP film in this study.

**Fig. 5:**
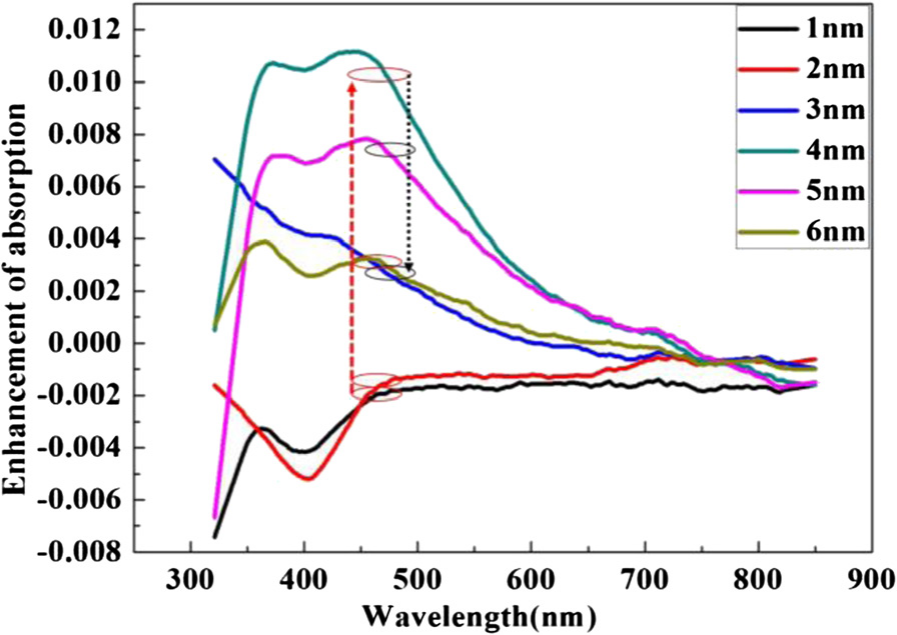
The absorption of devices with different thickness of Ag NP film

To analyze the effectivity of Ag NP film, the absorption spectra of multilayer (NPB/Alq_3_/PBD/Ag) with different thickness of Ag NP film were measured. Figure 5 shows the measured absorption spectra of the devices. It is clear that the 4-nm Ag NP film shows the highest absorption at 460 nm.

**Fig. 6:**
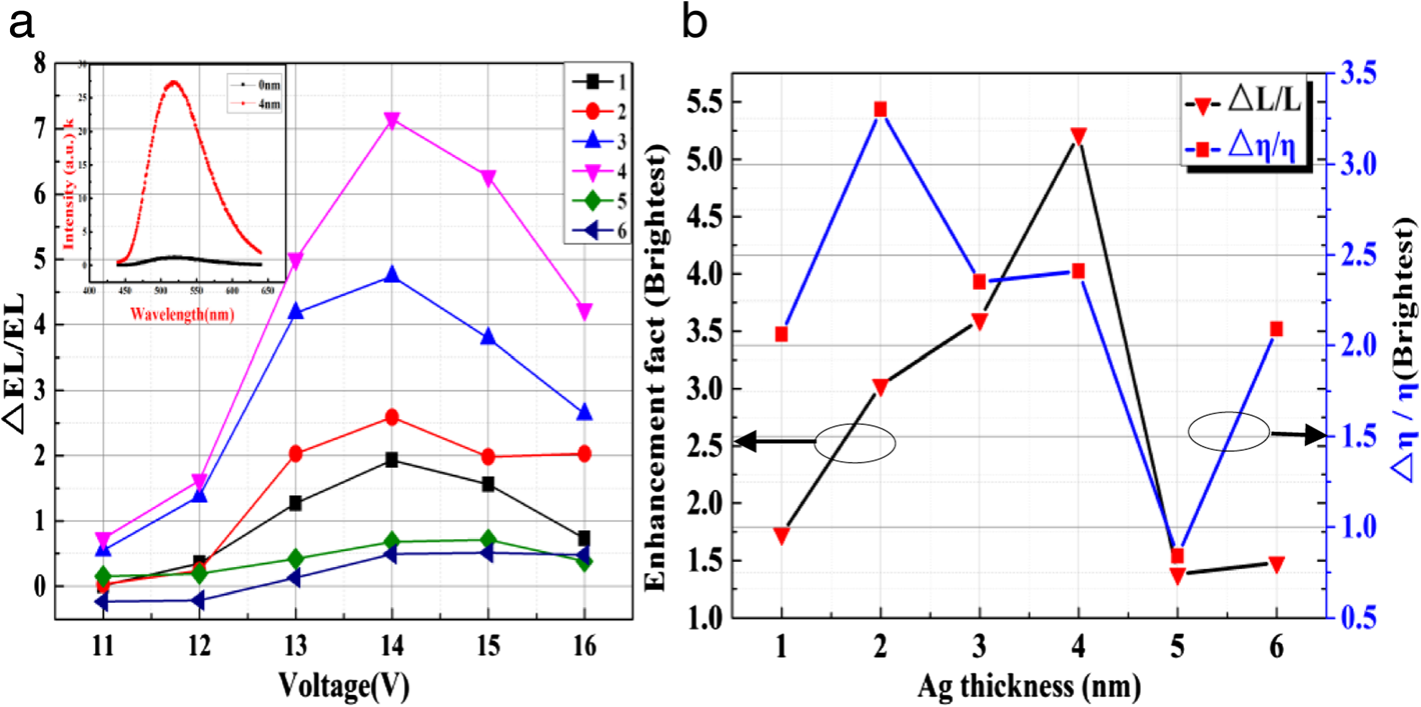
The enhancement of EL (**a**), luminance (**b**), and REQE (**b**) of devices

Figure 6a shows the enhancement of EL with different thickness of Ag NP films. The inset is the EL spectra of the devices with and without 4-nm-thick Ag NP film. We can see that the photon count intensity of EL is much greater than that without Ag NP films. The enhancement of photon count intensity increases with the thickness of Ag NP film. There is a significant increase when the thickness of Ag NP film is increased from 3 to 4 nm. The photon count intensity of the device can reach to 23 times than that of the device without Ag NP film. Figure 6b is the enhancement factor of luminance and relative external quantum efficiency (REQE) of the devices with different thickness of Ag NP films. As shown in the figure, when the thickness of Ag NP films increases, the enhancement factor increased until it reaches a maximum and then it decreases. The optimum luminance is obtained by the 4-nm Ag NP film; the maximum enhancement factor is about 5.4. The enhancement curve of REQE shows the same tendency with the enhancement of luminance. The highest REQE is obtained by the 2-nm Ag NP film. Combining the enhancement of luminance and REQE, we choose 4 nm as the optimal thickness of Ag NP film, whose REQE is about four times more than that of the device without Ag NP film.

**Fig. 7:**
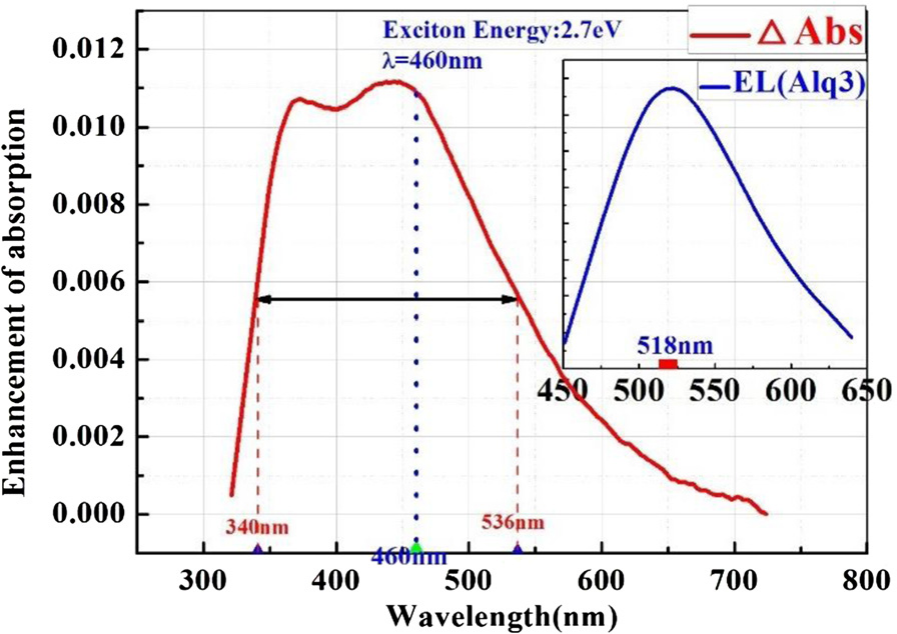
The absorption spectrum of Ag NP film

We measured the absorption of Ag NP film. Figure 7 is the absorption spectrum of Ag NP film. The inset is EL spectrum of Alq3 (emission peak is 518 nm).

**Fig. 8:**
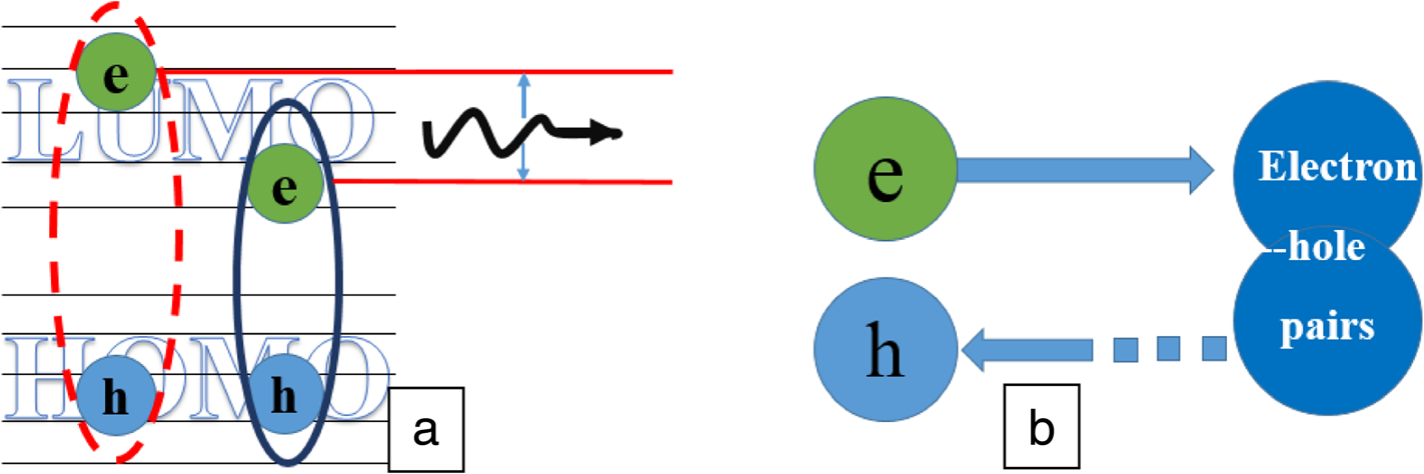
The electron-hole release energy (**a**) and would be balance (**b**)

Figure 7 presents that the absorption peaks of Ag NP are 373 and 443 nm. There is a strong LSPR area from 340 to 536 nm (we call it the action area). What surprised us was that the action area overlaps not only the emission frequency but also the electron-hole pair energy. As we know, electron-hole pair energy (energy gap between lowest unoccupied molecular orbital (LUMO) and highest occupied molecular orbital (HOMO)) of Alq3 is 2.7 eV (460 nm). The energy of electron-hole pair close to the resonance peak of Ag NP film induced new energy coupling ways and excited LSPR. When the OLEDs device is excited, electron and hole form electron-hole pair at first. The electron-hole pair is unstable. It releases energy by nonradiative relaxation and becomes an exciton (Fig. 8a). At the same time, the excited localized surface plasmons (LSPs) would transfer the energy to the exciton for light emission. Any change would break the balance (Fig. 8b).

In Fig. 7, we can see that LSPR area covers the energy of electron-hole pair. So once the pair forms, it would excite LSPs and resonance with it. The electron-hole pair would decrease, which breaks the balance. The formation of electron-hole pair would be promoted and the exciton number would increase. When the Ag NP is inserted into the OLEDs, the value of electron and hole increases.

**Fig. 9:**
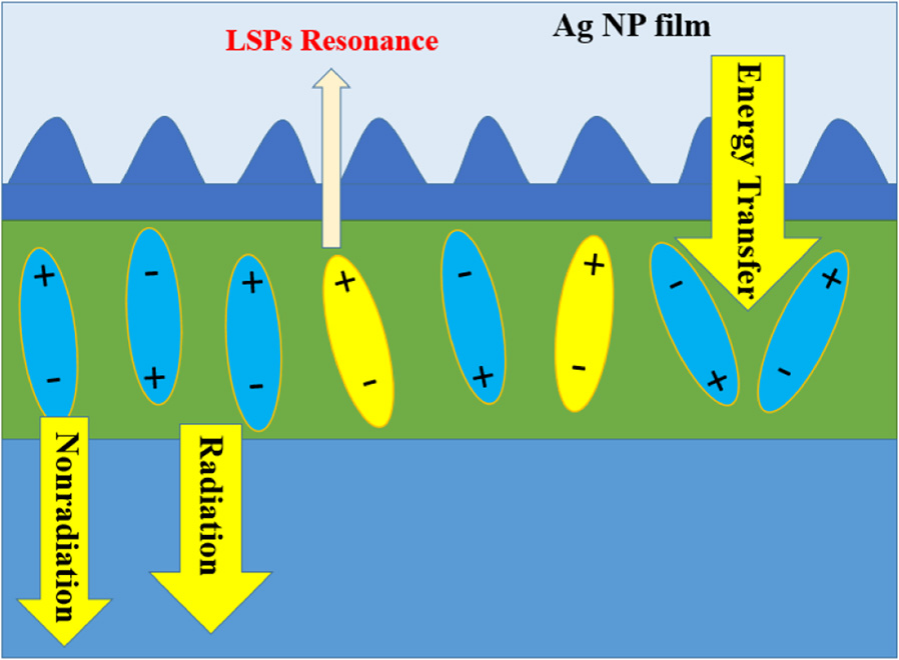
The interaction of electron-hole pair, exciton, and LSPs

Figure 9 presents the interaction of electron-hole pair, exciton, and LSPs. The electron-hole pair releases energy, which excites LSPs. They become an exciton (metastable state). Then, the exciton would release energy through radiative and nonradiative channels. Because the absorption spectra of Ag NP film also overlap with the emission light (emission peak is at 518 nm), there would be a strong interaction between the exciton and LSPR.

As we know, the lifetime for a free emission of an excited fluorophore can be expressed as

1 $$ {\tau}_0=\frac{1}{\varGamma_{\mathrm{RE}}+{\varGamma}_{\mathrm{NRE}}} $$

where Γ_RE_ is the radiative decay rate and Γ_NRE_ is the nonradiative decay rate. The introduction of Ag NP film would affect Γ_RE_ and Γ_NRE_ by inducing the additional energy transfer channel. Thus, the lifetime of fluorophore should be [17]

2 $$ {\tau}_0=\frac{1}{\varGamma_{\mathrm{RE}}^{\prime }+{\varGamma}_{\mathrm{NRE}}^{\prime }} $$

**Fig. 10:**
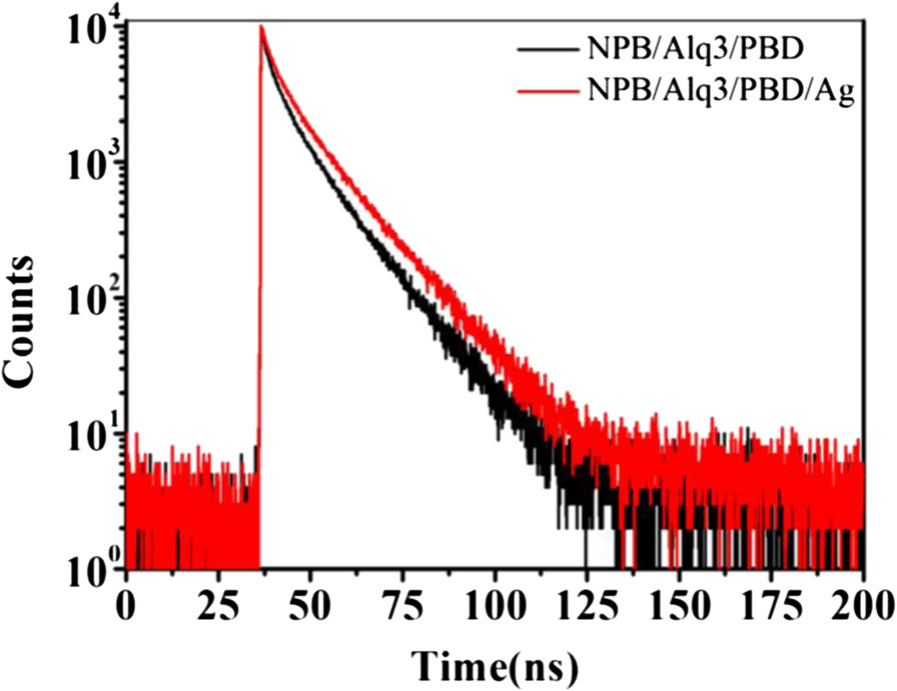
The PL decay kinetics of Alq3 with and without Ag NP film

To further investigate the relaxation of energy from the electron-hole pair, the PL decay of the device was measured. The decay kinetics of 518 nm for the samples with and without Ag NP film was analyzed. The kinetics (excited at 405 nm) was displayed in Fig. 10. The decay curve was fitted well by a biexponential function: *k*exp(*−t/τ*_1_) + (1−*k*) exp(*−t/τ*_2_)*.* Table 1 presents the fitted parameters of samples, NPB/Alq3/PBD and NPB/Alq3/PBD/Ag.

**Table 1:** The parameter of the decay kinetics of NPB/Alq3/PBD and NPB/Alq3/PBD/Ag

	*τ*_1_ (nm)	*τ*_2_ (nm)	*k*
Ag NP film(0 nm)	3.28	11.68	1.181
Ag NP film(4 nm)	3.68	13.10	1.156

We can see that the lifetime of Alq3 increased because of the insertion of Ag NP film.

The Γ′_RE_ increases due to the enhancement of luminescence. According to Eq. (2), we can deduce that the nonradiative decay rate Γ′_NRE_ decreases; moreover, the decreasing extent of the nonradiative decay rate is greater than the increasing extent of the radiative decay rate. Thus, the insertion of Ag NP film can suppress the nonradiative energy loss. The decrease of nonradiative decay rate reveals that the electron-hole pair transfers the energy to Ag films, which boosts the forming of electron-hole pair and the efficiency of OLED is increased.

The internal quantum efficiency (Γ_IQE_) from the device is given by radiative decay rate (Γ_RE_) and nonradiative decay rate (Γ_NRE_):

3 $$ {\varGamma}_{\mathrm{IQE}}\kern0.5em =\kern0.5em \frac{\Gamma_{\mathrm{RE}}}{\Gamma_{\mathrm{RE}}+{\Gamma}_{\mathrm{NRE}}} $$

The Γ_IQE_ would be affected because of the interaction with LSPs [18, 19]

4 $$ {\Gamma}_{\mathrm{IQE}}^{\prime }=\frac{\Gamma_{\mathrm{RE}}^{\prime }}{\Gamma_{\mathrm{RE}}^{\prime }+{\Gamma}_{\mathrm{NRE}}^{\prime }} $$

Equation (2) presents that the (Γ′_RE_ + Γ′_NRE_) decreased and the Γ′_RE_ increased. It suggests that the Γ′_IQE_ as in Eq. (4) should be increased.

## Conclusions

In summary, we have demonstrated the enhancement of OLEDs by inserting the Ag NP film. The enhancement of photon count intensity can reach to 23 times than that of the device without Ag NP film, and the maximum enhancement factor is about 5.4 when the Ag NP film is 4 nm. With inserting the Ag NPs, the energy would transfer to the LSPs from the electron-hole pair, which promoted the formation of electron-hole pair and excited LSPR. On the other hand, the emission peak also lays in the action area, where there would be a strong interaction between the exciton and LSPR. The lifetime measurement shows that Ag NPs increase the lifetime of Alq3, which indicates that the LSPR suppresses the nonradiative decay rate.

## Abbreviations

AFM: atomic force microscope
EL: electroluminescence
HOMO: highest occupied molecular orbital
L-MBE: laser molecule beam epitaxy system
LSPR: local surface plasmon resonance
LSPs: localized surface plasmons
LUMO: lowest unoccupied molecular orbital
NPs: nanoparticles
OLEDs: organic light emission diodes
PL: photoluminescence
REQE: relative external quantum efficiency
SPR: surface plasmon resonance
